# Understanding the epidemiology of iNTS disease in Africa in preparation for future iNTS- vaccine studies in endemic countries: Seroepidemiology in Africa of iNTS (SAiNTS) Study Protocol: Malawi site [Version 9.0]

**DOI:** 10.12688/wellcomeopenres.18054.2

**Published:** 2024-08-21

**Authors:** Helen Dale, Esmeda Chirwa, Priyanka Patel, Georgina Makuta, Felistas Mwakiseghile, Theresa Misiri, Innocent Kadwala, Maurice Mbewe, Happy Banda, Niza Silungwe, Kenneth Chizani, Paul Kambiya, Marc Henrion, Neil French, Tonny Nyirenda, Melita Gordon, Vacc-iNTS

**Affiliations:** 1 Institute of Infection, Veterinary and Ecological Sciences, University of Liverpool, Liverpool, Merseyside, L69 3GF, UK; 2 Clinical Research, Malawi-Liverpool-Wellcome Clinical Research-Program, Blantyre, Southern Region, PO Box 30096, Malawi; 3 Pathogy Department, Kamuzu University of Health Sciences, Blantyre, Southern Region, Private Bag 360, Malawi

**Keywords:** iNTS; immunoepidemiology; susceptibility; malaria; children, non-typhoidal salmonella

## Abstract

**Background:**

Non-typhoidal Salmonella (NTS) are a major cause of bloodstream infections amongst children in sub-Saharan Africa. A clear understanding of the seroepidemiology and correlates of protection for invasive NTS (iNTS) in relation to key risk factors (malaria, anaemia, malnutrition) in children in Africa is needed to inform strategies for disease control including vaccine implementation.

**Methodology:**

The SAiNTS study is a prospective community cohort study with paired serology samples from 2500 Malawian children 0–5 years at baseline and three months to measure age-stratified acquisition of lipopolysaccharide (LPS) O-antigen antibody (IgG) and serum bactericidal activity to the main serovars causing iNTS ( *Salmonella* Typhimurium and *S*. Enteritidis). Children are selected from mapped and censused randomly selected households in Chikwawa, Malawi; an area with substantial malaria burden. The sampling framework is set within a malaria vaccination (RTS,S/ AS01) phase 4 cluster randomized trial, known as the Epidemiology Study of Malaria Transmission Intensity (EPI-MAL), allowing exploration of the impact of malaria vaccination on acquisition of immunity to NTS. Risk factor data for invasive disease will be collected using rapid diagnostic tests for malaria and anaemia, anthropometry for malnutrition, and a validated questionnaire for indicators of socioeconomic status, water and sanitation. All data will be recorded through electronic case report forms using the REDCap and the Open Data Kit (ODK) platforms. Stool sample analysis includes NTS culture and pan-Salmonella polymerase chain reaction to assess enteric exposure and biomarkers of environmental enteric dysfunction. Cases with iNTS disease will be followed up for comparison with community controls.

**Conclusions:**

The final cohort of 2500 children will allow investigation into the impact of risk factors for iNTS on the acquisition of immunity in children 0–5 years in an endemic setting, including comparisons to partner seroepidemiology studies in three other sub-Saharan African sites (1000 children per site). The data generated will be key to informing iNTS disease control measures including targeted risk factor interventions and vaccine implementation through investigation of correlates of protection and identifying windows of immune susceptibility in at-risk populations.

## Introduction

Invasive Salmonella infection (both typhoidal and non-typhoidal serovars) are a major cause of mortality and morbidity worldwide ^1^. Non-typhoidal strains of Salmonella (NTS) are an important cause of enterocolitis, with an estimated disease burden of 95 million cases in 2017, but with a low case-fatality rate (CFR). However, non-typhoidal salmonella infections can be invasive, causing bacteraemia, meningitis and other focal infections. Cases, predominantly in children under five years of age, present with non-specific febrile illness and have a high CFR ^2^. Invasive NTS (iNTS) disease is also associated with poor nutritional status, younger age, immune-suppression and severe malarial anaemia ^1^. Invasive NTS is most commonly seen in Sub-Saharan Africa (SSA), where certain serovars are endemic within populations.

Progress in controlling iNTS infections are hampered by non-specific clinical presentation, lack of accurate diagnostics, and incomplete knowledge of transmission pathways within the community and correlates of protection.

### Vaccine development

There is no currently licensed vaccine available against iNTS disease. Three iNTS vaccines are now in clinical stages of development. One is a trivalent lipopolysaccharide-flagellin conjugate, targeting typhoid and the two commonest iNTS serovars in Africa ^3^. A second is another trivalent conjugate vaccine developed by the International Vaccine Institute (IVI) and supported by Wellcome Innovations ^4^. The third is a novel parenteral bivalent iNTS vaccine based on Generalized Modules for Membrane Antigen (GMMA), also targeting serovars Typhimurium and Enteritidis ^5^.

***The VacciNTS consortium – accelerating a novel iNTS vaccine***

**Figure 1.**
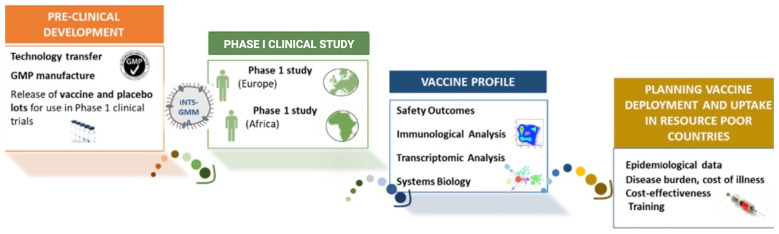
Overall Vacc-iNTS strategy.

GMMA has a unique and robust technical production platform offering high yields at a low cost as compared to other iNTS vaccines. The VacciNTS consortium has recently been funded by a European Union H2020 grant and aims to accelerate WHO prequalification and licensure of this vaccine and vaccine deployment in SSA where the burden of iNTS disease is greatest ( Figure 1). The highly cost-effective GMMA-technology is based on outer membrane blebs released by genetically modified bacteria. Supporting activities include cross-sectional sero-epidemiological studies in potential future phase 2 and 3 clinical trial sites in Malawi, Ghana, Burkina Faso and Kenya (WP7); assessment of the global burden of disease using different risk-based modelling methodologies, and stakeholder engagement and advocacy activities (WP8); and assessments of the cost of iNTS illness and cost-effectiveness of iNTS vaccine introduction (WP9). GSK Vaccines Institute for Global Health (GVGH) are providing the laboratory training and support for the sero-epidemiology studies.

***VacciNTS study sites***

Malawi; The rural district of Chikwawa, Southern Malawi, has high seasonal rainfall, humidity, and temperature, and low altitude. Most of the population rely on subsistence farming and over half of households have food insecurity. It is the site of the Epidemiology Study of Malaria Transmission Intensity (EPI-MAL) RTS,S/AS01 malaria vaccine cluster randomised trial, and FIEBRE fever surveillance study.

Burkina Faso; Two sites: 1. Polesgo and 2. Nioko II informal settlements in the Nongr-Massom health district, which are both part of the Ougadougou Health and Demographic Surveillance System (HDSS), and passive fever surveillance for Salmonella since 2011.

Kenya; Three sites: 1. Mukuru Slums (informal settlements), 20km East of Nairobi, 2. Lwak mission hospital (rural, agricultural population), Kisumu County, Western Kenya, 3. Kilifi County Hospital (KCH) rural population part of Kilifi HDSS.

Ghana; Two sites: 1. Agogo Presbyterian Hospital (APH), Asante Akim North Municipal district largest referral hospital in the district serving semi-urban and rural populations. 2. Komfo Anokye Teaching Hospital (KATH) and the Maternal and Child Hospital (MCHH) Kumasi (second largest city in Ghana), Ashanti region. KATH serves as a referral hospital for the northern part of Ghana.

A separate core-protocol ^6^ (Extended data) has been written for all sites. This article will focus on the Malawi site protocol due to more complex study design.

***Immune correlates of protection on the pathway to vaccine licensure***

The gold standard for vaccine licensure and policy decisions are double-blind placebo-controlled randomized trials to determine vaccine efficacy with clinical endpoints. However, this can be prohibitively expensive and a major logistic undertaking requiring very large sample sizes if the clinical endpoint is uncommon and expensive to diagnose, as is the case for iNTS. Immune correlates of protection (CoP) can accelerate the development of vaccines, providing non-clinical pivotal end points in vaccine studies ^7, 8^. CoP can be defined as immune markers shown to correlate statistically with a reduced risk of disease ^9^.

Estimating an easily measured, robust antibody CoP would be a key step in accelerating any iNTS vaccine towards licensure and deployment.

### Immunological basis for disease

***Immunological susceptibility and risk-factors for iNTS disease***

**Figure 2.**
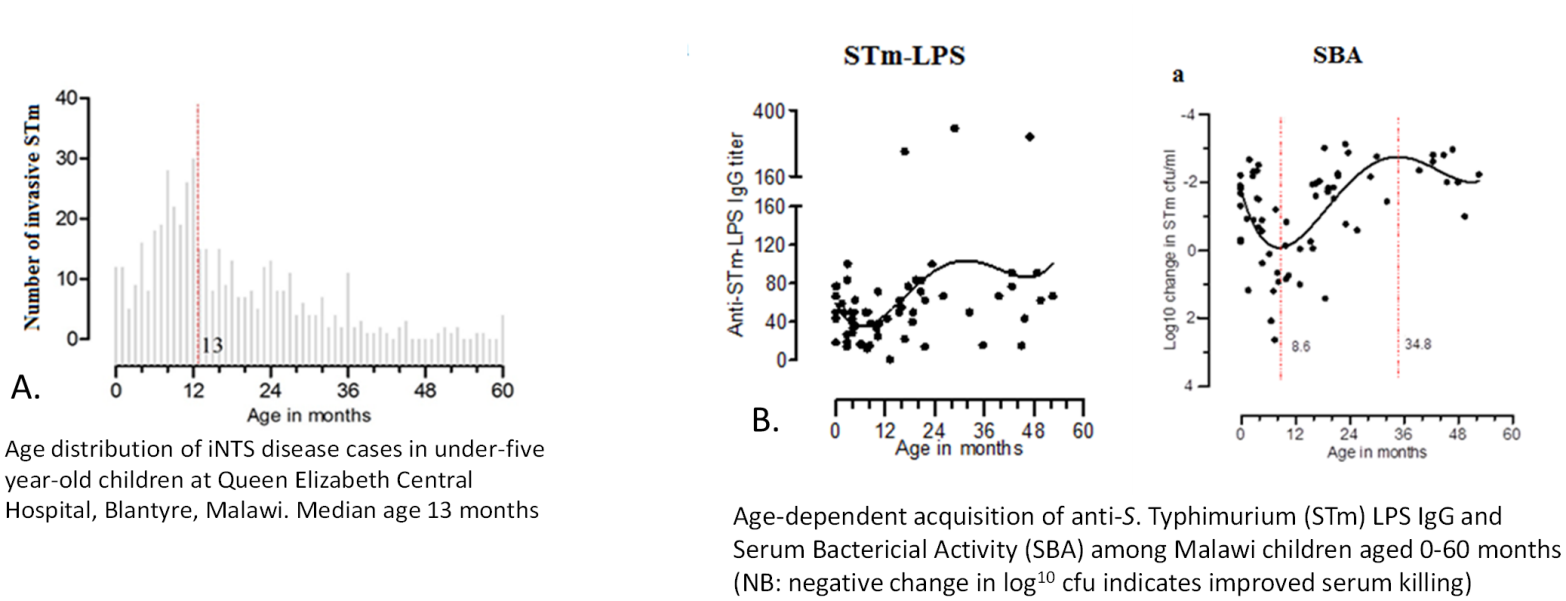
**A**: Age distribution of iNTS cases (0–60 months). **B**: Age distribution of anti S. Typhimurium LPS antibodies ^11^.

iNTS disease has a bimodal age distribution in SSA, being commonest in adults with HIV, and in young children ^10^, among whom the median age of invasive disease is 13 months ( Figure 2A and 2B) ^11^. iNTS disease is linked to several underlying susceptibilities among children, particularly recent malaria, severe malarial anaemia and malnutrition ^12^. Children between the ages of six months to two years are particularly at risk, suggesting protection before six months by breastfeeding and transferred maternal antibody ^13, 14^.

***The acquisition of natural immunity to iNTS***

**Enteric exposure to iNTS**

Systemic natural protective immunity to iNTS, which is largely acquired between six months - three years in Malawi, develops following exposure of the gastrointestinal (GI) tract to Salmonella. A longitudinal study of a cohort of 50 healthy Malawian children, who were sampled monthly, showed that between the ages of six-18 months more than 50% had at least one episode when a non-typhoidal strain of Salmonella was detected in their stool. Most of these episodes were not associated with current or recent diarrhoea. In any month, there was an 8–9% chance of stool positivity for Salmonella. Quantitative PCR using a pan-Salmonella marker (TTR, tetrathionate reductase gene) was more sensitive than culture for detection ^15^. Among samples that were culture-positive, 48% of isolates comprised ST313 *S. *Typhimurium, with the remainder being a wide range of non-typhoidal serovars that are not seen from blood cultures in Malawi. We have termed these enteric infection/exposure events “eNTS” events (unpublished work T. Nyirenda PhD Thesis: https://livrepository.liverpool.ac.uk/2010084).

**The acquisition of natural systemic immunity**

We have described the age-stratified sequential acquisition of CD4 cellular immunity, followed by functional antibody-mediated immunity and serum bactericidal activity (SBA) against iNTS among children in Malawi ^11^. This demonstrated the nadir of functional humoral immunity, which occurs after the waning of maternal antibody, but before the sequential acquisition of first cellular immunity, then anti-LPS IgG antibody and associated functional SBA. This nadir coincides with the peak incidence of iNTS disease among children at nine-13 months, further demonstrating how a serological protective threshold of humoral protection develops following eNTS exposure over time.

Robust defence against iNTS requires both cellular and humoral immunity, and we have previously demonstrated both cellular and humoral defects that cause susceptibility to iNTS disease. Humoral and cellular defences have both been shown to be impaired in current and recent uncomplicated malaria ^16^. Bound, complement-opsonised antibody is required both for SBA, and for uptake of bacteria by phagocytes prior to killing by respiratory burst.

There are critical current knowledge gaps in our understanding of the relationship of enteric Salmonella exposure and the development of protective immunity amongst those in the highest risk age bands; of what are the correlates of protection for iNTS disease; and of how the effect of known susceptibility risk factors in different populations affect these relationships.

Several mechanisms of humoral and cellular immunity are required for protection against iNTS disease, and different risk factors act on different elements of the immune response. This means that some of the resultant background immunological susceptibilities might be fully vaccine-responsive (e.g. a lack of sufficient protective antibody), while others might not be fully ameliorated by vaccine-induced immunity (e.g. periods of complement deficiency, or failure of respiratory burst due to haemosiderin damage to phagocytes). This means that the potential impact of vaccine deployment to prevent iNTS disease might be different in different epidemiological settings.

Understanding these influences will ultimately allow us to understand the effects of specific known risk factors, and to describe antibody correlates of protection. This will allow us to accelerate vaccine licensure and deployment, and to model predicted vaccine impact among different susceptible populations in SSA.

### Model of exposure and immunity

**Figure 3.**
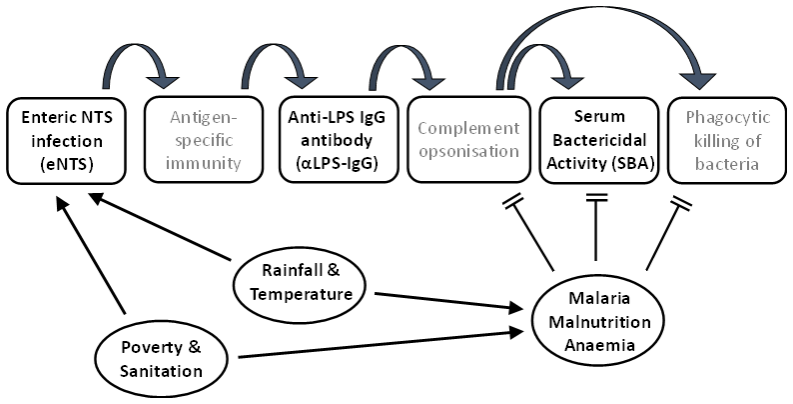
Theoretical framework for a model of exposures and protection in iNTS disease.

We therefore present a theoretical framework ( Figure 3) for asymptomatic enteric infection with NTS (eNTS) among Malawian children, followed by the development of first antigen-specific cellular immunity, and then functional systemic immunity. Systemic immunity is evidenced by the presence of anti-Salmonella LPS IgG antibody (αLPS IgG) which, when combined with complement opsonisation, results in functional protection, reflected in SBA. This is likely to be influenced by several factors, including social factors such as poverty and sanitation, by seasonal climatic conditions through the year, and by the impacts on immune status of malaria, malnutrition and anaemia. There is likely an interaction between several of these influencing risk factors.

***OptiVaNTS-Optimising Vaccination for iNTS disease in Africa***

Serum samples from SAiNTS will be used for systems serology analysis to map and define the mechanistic humoral correlates of immunity to invasive NTS.

### Contributing studies

***Malaria (RTS,S/ AS01) vaccine cluster randomised trial – EPI-MAL***

There is currently a phase 4 cluster randomised trial of the RTS,S/AS01 malaria vaccine being carried out in Chikwawa, Malawi, in which we will co-locate and embed the SAiNTS study. This offers a unique opportunity to explore the potential impact of the malaria vaccine on the development of the immune response to NTS, given that malaria has been shown to impair bactericidal activity (as described above).

### Nutrition and gut health

Myeloperoxidase (MPO) detected in stools is an indicator of intestinal inflammation and environmental enteric dysfunction, therefore understanding the association between MPO in stools and enteric NTS infections and acquisition of immunity to NTS can provide key insights into the mechanisms of disease susceptibility.

### Objectives

***Broad objective***

To understand the acquisition of immunity to NTS and epidemiology of eNTS in children 0–5 years, and how this immunity varies with risk factors (malaria, anaemia, malnutrition and sickle cell disease) and geographical setting.

***Specific objectives***

1.   To estimate the seroprevalence by age of NTS in children 0–5 years in Chikwawa, Malawi.

2.   To estimate the seroincidence by age of NTS in children 0–5 years in Chikwawa, Malawi.

3.   To explore the effect of the main risk factors: malaria, malnutrition, anaemia, and sickle cell disease on the acquisition of humoral immunity to NTS (IgG and SBA).

4.   To describe the relationship between IgG and SBA including effect modification by age.

5.   To estimate the age-stratified seroprevalence of Salmonella enteric infections (eNTS) by risk factor group: malaria, malnutrition, anaemia, and sickle cell disease.

6.   To compare age-stratified acquisition of immunity to NTS across sub-Saharan African settings (Vacci-NTS sites: Kenya, Malawi, Burkina Faso, Ghana).

**Exploratory objectives**

1.   To define an antibody CoP based on the inverse relationship between known age-stratified clinical disease incidence and age-stratified seroprevalence.

2.   To investigate the impact of malaria (RTS,S/ AS01) vaccination on age-stratified acquisition of bactericidal activity against NTS.

3.   To investigate how intestinal inflammation/ environmental enteropathy relates to enteric NTS infections and development of immunity.

4.   Understand antibody kinetics of immune response to NTS and *Salmonella* Typhi in blood culture confirmed cases and community serosurvey samples to inform seroincidence estimates.

## Methods

### Study design

***Prospective community cohort***

We will carry out a prospective cohort study with paired serology samples at baseline and 3-months taken from the same individuals. Two thousand children aged 0–5 years sampled across age-strata will be recruited, randomly sampled throughout household-censused enumeration areas. We will spread sampling evenly throughout the year in Chikwawa district, Malawi, aiming to capture peak iNTS season in the middle of the study period. These children will be evenly recruited from the vaccinated (Montfort) and non-vaccinated clusters (Chikwawa) of the EPI-MAL trial. We will select households containing children 0–5 years via a two-stage sampling technique. In the first stage enumeration areas (EA) will be selected using probability proportional to size (PPS) sampling, with stratification according to malaria prevalence. In the second stage households containing children 0–5 years will be randomly sampled from selected Enumeration Area (EA). Any child eligible in the household will be invited to participate in the study.

We will collect approximately 2500 stool samples and 4000 paired blood samples (to account for 20% loss to follow-up), measuring antibody titres (IgG) and changes in serum bactericidal activity (SBA) for the most common NTS serovars (S. Typhimurium and S. Enteritidis) and other serovars, to understand the age-stratified acquisition of immunity and how this varies with risk factor exposure. We will perform anthropometric measurements, malaria (rapid diagnostic tests [RDT] and thick blood films), haemocue and sickle RDT to assess the association with the main risk factors. Any children found to have positive malaria tests will be provided treatment by the field team. Any child with a low haemoglobin will provided treatment according to the Integrated Management of Childhood Illnesses (IMCI)/ World Health Organisation (WHO) guidelines for management of anaemia ^17^ and referred to hospital for follow-up. A comprehensive record of the treatments administered throughout the study will be documented for subsequent analysis. Any child diagnosed with sickle cell disease or malnutrition (based on weight-for-height z-scores or Mid-Upper Arm Circumference (MUAC)) will be referred to the local health facility for further management.

Detailed immune and antigenic investigation of these samples will be carried out in collaboration with OptiVaNTS. An immune correlate of protection will be estimated using age-stratified acquisition of immunity and age-stratified incidence of clinical disease defined by routine blood cultures collected through MLW-Queen Elizabeth Central Hospital. Stool samples will be tested for salmonella culture and Polymerase Chain Reaction (PCR), which will enable us to model the effect of eNTS at visit 1 to induce systemic immunity at visit 2. Data will be collected on sociodemographics and indicators of water, sanitation and hygiene (WASH) using the REDCap and Open Data Kit (ODK) platforms (see Table 1).

**Table 1.** Summary of data to be collected.

Biological measure	Variable	Target n observations	Methods
Enteric Salmonella infections (usually transient & asymptomatic)	eNTS event	4000	Stool culture in selective broth, DNA extraction and qPCR for TTR gene.
Serological marker of invasive NTS infection	iNTS event	4000	ELISA for anti-S. Typhimurium and S. Enteritidis LPS IgG, conducted on 3-month paired serum samples.
Functional protective measure of serum killing	Serum killing	4000	Serum bactericidal activity high-throughput assay conducted on 3-month paired serum samples.
Rainfall and temperature	Climate	475 days	Daily measurements by Malawi Meteorological Office.
Poverty and sanitation	Poverty	4000	Validate questionnaire of household wealth and WASH (water, sanitation, and hygiene).
Current or recent malaria	MRDT	4000	Point-of-care fingerprick lateral flow antigen detection Malaria Rapid Diagnostic Test (P. falciparum HRP2 detection).
Haemoglobin	Hgb	4000	Haemoglobin (g/dl) measured by point of care fingerprick haemocue test.

We will compare the age-stratified acquisition of immunity (IgG and SBA) between children receiving the malaria vaccine (RTS,S/AS01) and un-vaccinated children matched on factors known to influence vaccine uptake. The EPI-MAL study has conducted a GPS-mapped household census, allowing us to randomly select children across the study age-strata to approach for recruitment. Selection of enumeration areas has assumed approximately 50% uptake of consent to the study, and statistical power calculations have assumed a further 10% drop out for the second visit.

**Figure 4.**
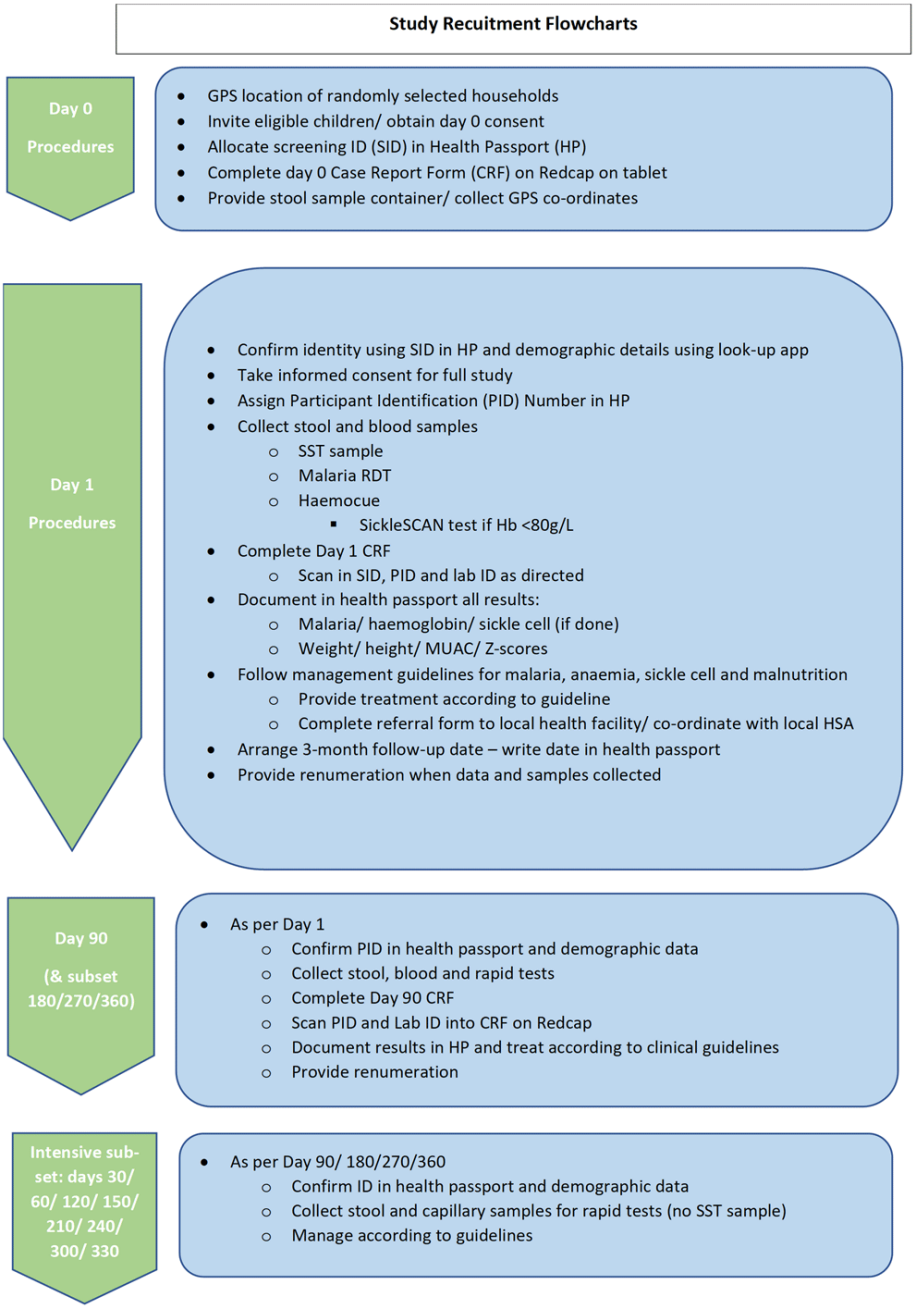
Study recruitment summary flowchart (see supplementary documents for detailed flowcharts).

Detailed schemas illustrating the rotation of field teams recruitment and sampling of stool and blood are shown in Table 2 & Figure 4 also see full protocol ( *Extended data*) ^6^.

**Table 2.** Detailed schema of SAiNTS field illustrating teams 3-monthly rotations through sequential enumeration clusters.

		**Q1, 2021**								**Q2, 2021**								** Q3, 2021**								**Q4, 2021**								**Q1, 2022**									**Q2, 2022**			
		**V1 C1**									**V2 C1**									**V2 C2**									**V2 C3**									**V2 C4**								
											**V1 C2**									**V1 C3**									**V1 C4**																	
**RTS,S unvaccinated**	1																																													
High malaria	2																																													
prevalence	3																																													
(Chikwawa)	4																																													
N=1200	5																																													
	6																																													
	7																																													
	8																																													
	9																																													
**RTS,S vaccinated**	10																																													
Low malaria	11																																													
prevalence	12																																													
(St Montfort)	13																																													
N=1200	14																																													
	15																																													
	16																																													
	17																																													
	18																																													

Scheme showing sampling rotation through enumeration areas (EAs) (1 to 18), with four cohorts (C1 to C4) starting at 3 monthly time intervals in each of the high and low malaria transmission areas. Each EA will be visited at four time periods throughout the year, to capture variations in seasonality. A total of 2400 children will be recruited, 1200 at each site, with 30-40 children recruited over 7-12 days at each EA at each round of recruitment. Each child will have paired samples collected 3 months apart, and the next cohort of children will be recruited at the same time as the day 90 follow-up at each EA.

### Longitudinal cohort and weekly stool sampling

A subset of children will continue to have extra follow-up visits with blood samples at six, nine and 12 months to track individual immunity and waning over time. In addition, this subset will have additional monthly stool samples and malaria RDTs to explore the extent of missing exposures which may have an impact less than three months on humoral immunity, and the impact of repeated exposures on acquisition of immunity. We will carry out weekly follow-ups for a subset of children with *Salmonella* stool culture up until they have two consecutive negative stool cultures, to assess the duration of asymptomatic *Salmonella* carriage.

***Case-control study***

We will prospectively recruit any child between the ages of 0–5 years with a positive blood culture for NTS at Queen Elizabeth Central Hospital (QECH), Zingwangwa and Ndirande health centres. Participants will have a blood sample to test for immune markers as close as possible to presentation, as soon as blood culture is determined to be positive, and repeat samples at one, three-, six-, nine- and 12-month follow-ups. These samples will be tested for antibody and bactericidal activity to NTS and other immune markers to understand acquisition of immunity post-invasive disease compared to immunity developed in the community sero-survey data. All other details including risk factor testing and socio-economic and WASH questionnaires will be carried out in the same way as the main study. For each case, a control from the community serological survey data from SAiNTS samples will be recruited and matched on age and geographic location (as close as possible) to compare immune markers at baseline and post-invasive disease to help validate estimated correlates of protection.

Blood culture positive cases of *Salmonella* Typhi will also be recruited and have a blood sample taken at presentation at one, three, six, nine and 12 months to measure antibody kinetics for immune markers for acute typhoid infection, to inform models to estimate seroincidence of typhoid infections from future community serosurveys. Stool samples will also be collected at each visit. The stool will be tested for *Salmonella* and *E. coli* and other microbes associated with health and disease which can be found normally in healthy children and to look at the health of the gut. Additionally, information on antibiotic usage in the past month will be collected from both the confirmed blood culture case and members of their household.

***Serosurveys in other sub-Saharan African sites***

As part of the VacciNTS sero-epidemiology work-package three other field (Kenya, Burkina Faso and Ghana) sites have been chosen for the sero-epidemiological surveys. A core protocol has been written for all sites (see Vacc-iNTS Core protocol for details of study methodology for other sites ( *Extended data* ^6^)). Malawi will be the central site for this project, leading on data management and laboratory training for other sites – with support from GVGH. A minimum of 1000 children will be recruited from each of the 4 sites (Malawi, Kenya, Burkina Faso and Ghana). In Malawi we have planned to expand the sample size to 2500 and to take paired samples 3 months apart with additional meta-data on iNTS risk factors. The other sites will not test for risk factors, or use RTS,S/AS01 vaccine study sites. All serological samples will be tested for IgG and SBA for NTS, however, there is no obligation for the other sites to test for risk factors.

### Study population

***Prospective community cohort and longitudinal cohort***

Guardians of children aged 0–5 years resident in Chikwawa district enumeration areas defined by the EPI-MAL trial will be approached to enter the study.

**Inclusion criteria**

-   Child ≤ 5 years

-   Planning to be in study area for next three months

**Exclusion criteria**

-   Not resident in the catchment area

-   Deemed clinically unstable by the survey team

***Case control study***

We will prospectively recruit any child between the ages of 0–5 years with a positive blood or cerebrospinal fluid culture for NTS identified through the Malawi Liverpool Wellcome (MLW) Programme’s Laboratory Information Management System (LIMS) as a case.

**Inclusion criteria (iNTS Cases)**

-   Blood or cerebrospinal fluid culture positive for non-typhoidal salmonella

-   Child ≤ 5 years

### Study period

The study is planned to be carried out over three years from proposal to dissemination of findings (see Gantt chart [ Table 3] outlines the timing of planned study activity and quarter of completion.

**Table 3.** Gantt chart of planned study activities.

Activity	2020						2021												2022												2023					
Year and Quarter	Q3			Q4			Q1			Q2			Q3			Q4			Q1			Q2			Q3			Q4			Q1			Q2		
Rainy season																																				
NTS peak-season																																				
Ethics & governance																																				
SOP’s, data tools & validation																																				
Public & community engagement																																				
Stakeholder engagement																																				
Fieldwork test-pilot																																				
Field sampling cohort 1																																				
Field sampling cohort 1&2																																				
Field sampling cohort 2&3																																				
Field sampling cohort 3&4																																				
Field sampling cohort 4																																				
Lab analyses in real-time																																				
Lab analyses 4000 stored samples																																				
Data cleaning																																				
Primary Mathematical modelling																																				
Secondary Mathematical modelling																																				
Analysis across all VacciNTS sites																																				
Data write-up, presentation																																				
Feedback to community & stakeholders																																				

### Sample size

 All code can be found here ( *Extended data* ^18^).

***Sample size simulations for serosurvey***

**Figure 5.**
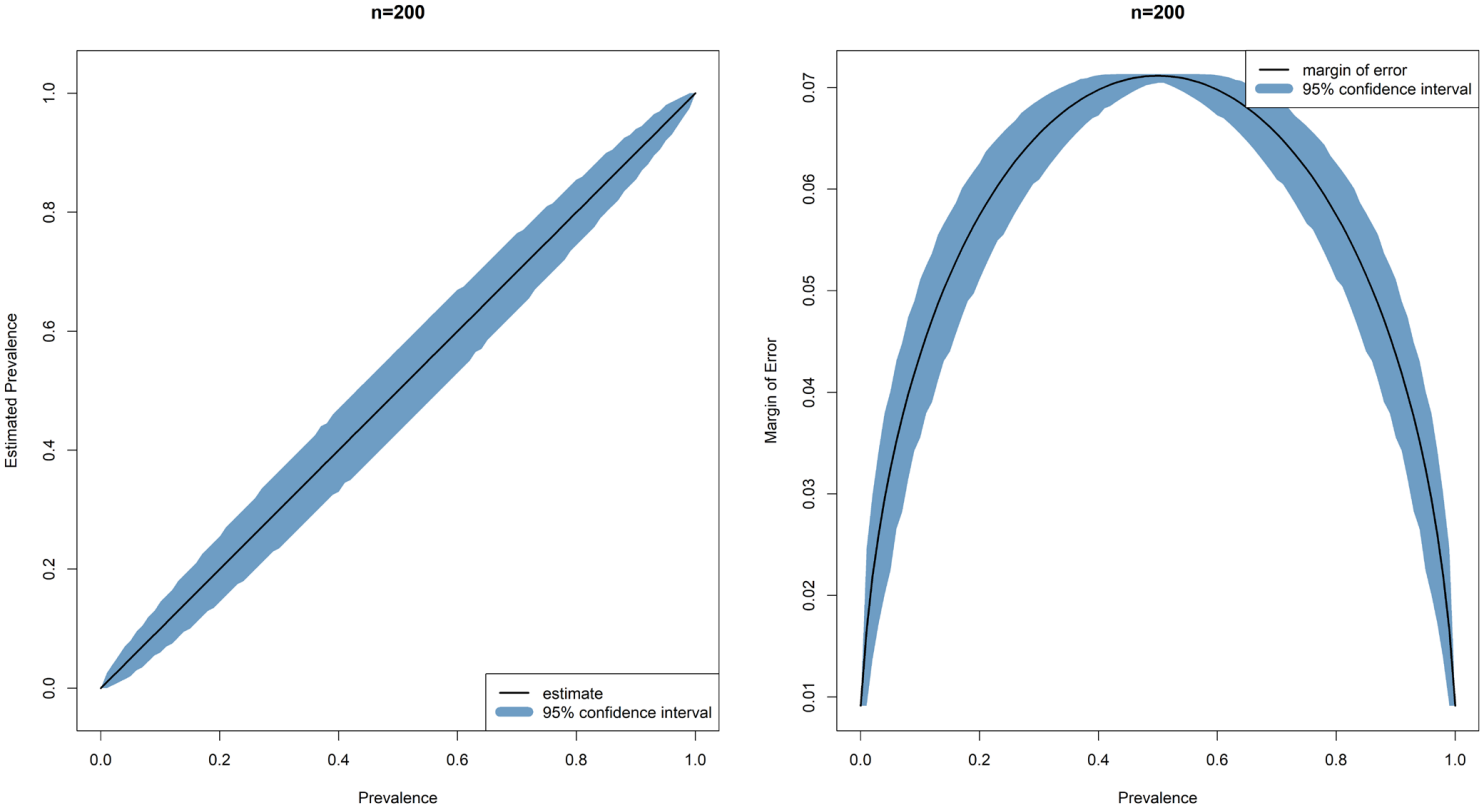
Margin of error and 95% Confidence Intervals across the range of seroprevalences.

Simulated data (100,000 simulations) demonstrate an acceptable margin of error and confidence interval for estimation across all seroprevalences for 1000 single samples (200 in each of five age groups, from 0–11, 12–23, 24–35, 36–47, 48–60 months), as a minimum sample size for each site in Malawi, Kenya, Burkina Faso, and Ghana ( Figure 5).

***Sample size simulations for community cohort (Malawi site only – additional analysis)***

Power for detecting change in seroconversion rates due to enteric salmonella exposure eNTS.

**Figure 6.**
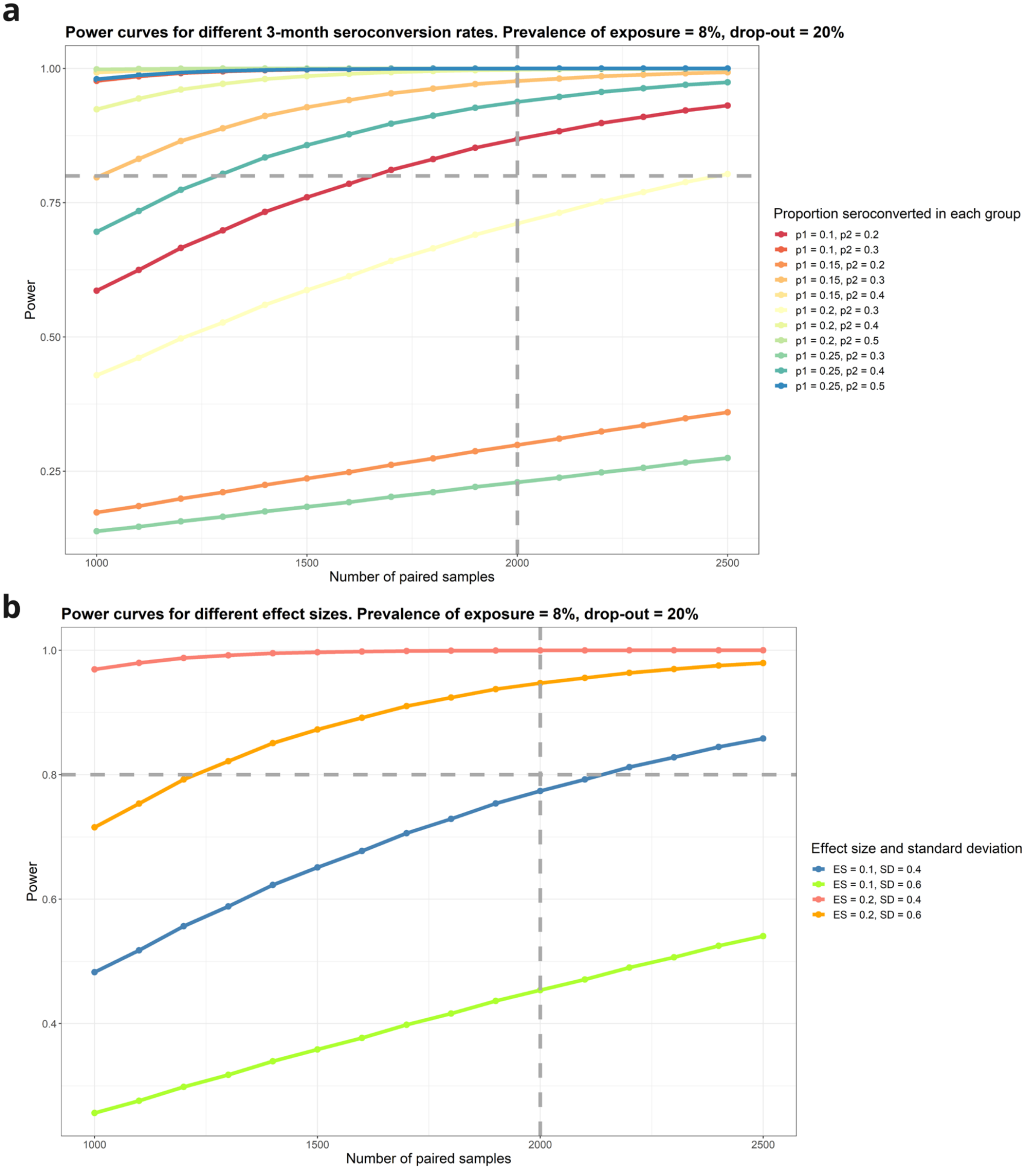
**A**: Power for detecting seroconversion following enteric infection. **B**: Power for detecting change in SBA following enteric salmonella exposure (8%) prevalence (N=2500).

Power was estimated for the change in seroconversion comparing proportions of unequal size between two groups (visit 1 and visit 2). A range of scenarios are computed around the initial proportion of individuals that are already seroconverted at the first sample and how many more are seroconverted at the second sample. Power is high for most scenarios, particularly when the effect of exposure results in a doubling or more of the seroconversion rate ( Figure 6A).

**Change in SBA due to eNTS**

Statistical power is high across a range of assumptions ( Figure 6B). With a sample size of 2,500 recruited individuals, 20% loss to follow-up, a difference of 0.2 in SBA between children with salmonella PCR-positive stools compared to those with negative stools, can be detected in the three-month follow-up visit sample with 99.97% power, assuming a standard deviation of 0.4 for SBA ( Figure 6B).

**Change in SBA in longitudinal cohorts**

Power is over 80% for a sample size of 20 in each arm for the case-community control longitudinal study (visits at 0,3,6,9,12 months) to detect a difference in SBA of 0.75. For the community cohort comparing SBA between malaria positive and negative children; 19 in each arm gives 80% power (Extended data ^18^).

**Changes in antibody titer by age**

**Figure 7.**
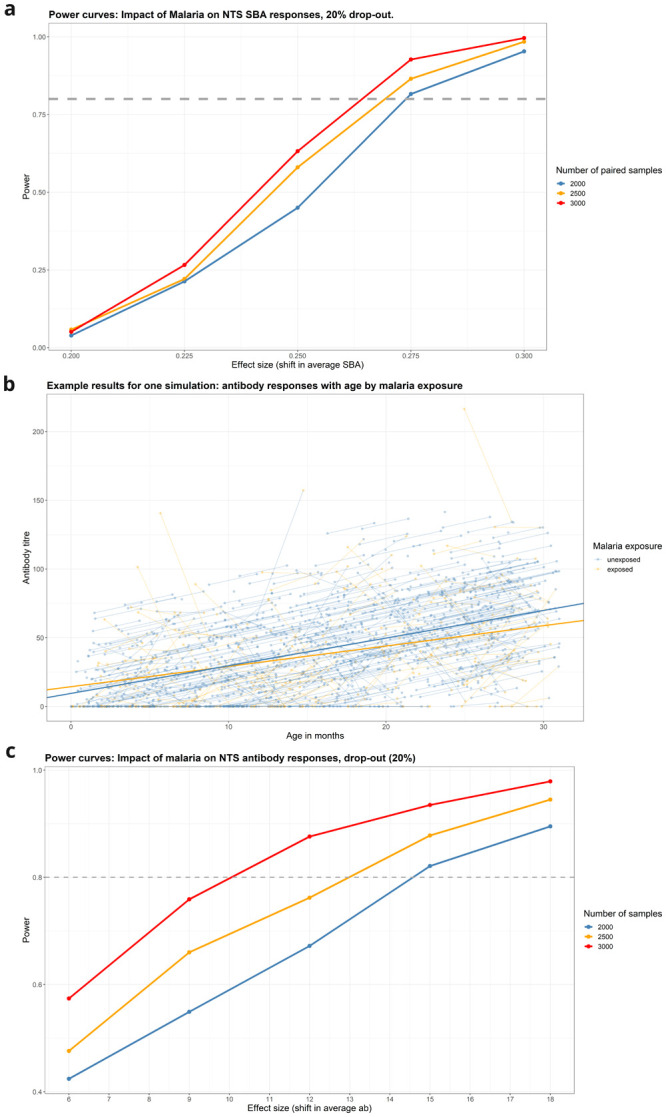
**A**: Power for detecting change in SBA due to malaria infection. **B**: Example of data from a single simulation (malaria). **C**: Power curves for detecting impact of anaemia on mean NTS antibody level.

 There is good power (>80%) to detect a difference of 13 antibody titer between visits for children with and without either malaria or anaemia with a sample size of 2500 ( Figure 7A–C). For malnutrition and sickle cell disease the changes in antibody titre between samples has good power (>80%) to detect a change in antibody titre of 47 and 68 respectively between visits for a sample size of 2500, which is much less sensitive than the other methods – therefore these will be exploratory endpoints ( Table 4).

**Table 4.** Summary of all sample size calculations carried out by risk factor and outcome.

Risk factor	Prevalence	Difference in SBA between 3 monthly samples (CFU/ml)	Difference in antibody titer	Difference in seroconversion over 3 months	Power to detect effect
*Malaria*	16%	0.25	13		>=80%
*Moderate to severe anaemia*	20%	0.225	13		>=80%
*Malnutrition*	2%	0.65	47		>=80%
*Sickle cell disease*	1%	0.9	68		>=80%
*Enteric NTS infection*	8%	0.2	15	15–20%	>=80%/ 99.97%
*Malaria RTS,S/AS01 vaccination*	60% coverage	1.5	-	-	>=80%

**Changes in serum bactericidal activity by age**

**Figure 8.**
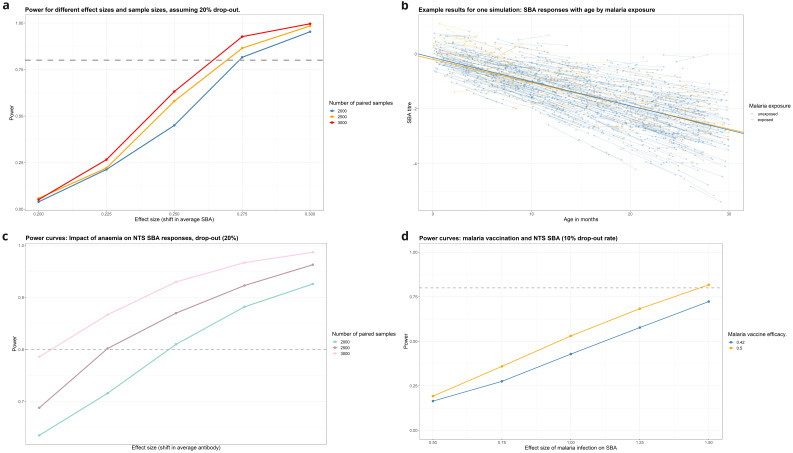
**A**: Power for detecting change in SBA due to malaria infection. **B**: Example data from a single simulation (malaria). **C**: Power for detecting change in SBA due to Anaemia. **D**: Power for detecting change in SBA due to malaria vaccination (N=2500).

With malaria prevalence of 16%, the simulations found that 2,500 paired samples would be well-powered (power >80%) to detect changes in SBA of 0.25 CFU/ml between three-monthly samples ( Figure 8A–B, Table 4). This change is equivalent to the gain in SBA activity naturally acquired over 2.5 months in the 9-36-month age-group, therefore lower than would be expected to be seen over a three-month period on average of natural acquisition of immunity. Simulations were run for other risk factors and protective factors including malaria vaccination ( Table 4 and Figures 8C and 8D).

### Community engagement

A community engagement plan will be developed in collaboration with the Science Communication Department (SciCom), MLW. Sensitive issues regarding for example blood and sample collection from young children, asking personal information about poverty and concerns and fears regarding risk of COVID-19 transmission and staff working in Personal Protective Equipment will all be addressed. SAiNTS-Malawi will utilise the existing infrastructure of public engagement activities that are planned and continuously implemented by SciCom throughout the year. These include Community Advisory Group (CAG), Chiefs, District Executive committee (DEC) and other stakeholders’ meetings, Science Cafes, etc. 

### Sample storage and analysis

All laboratory process have specific SOPs which are stored in the OSF folder ( https://doi.org/10.17605/OSF.IO/FUE3T ^6^).

***Serum sample collection and processing***

Serum samples were collected in serum-gel vacutainers and transported daily to the main laboratory at KUHeS in Blantyre in cooler boxes with temperature loggers, maintaining a temperature of 4–8°C. Subsequently, the samples were centrifuged, aliquoted and stored at -70°C (range -65°C to -80°C).

***Analysis of clinical sera by ELISA***

Assays developed by our collaborators at GVGH will be performed as per published protocols by Aruta *et al.*, 2023. Briefly, antibody titers to OAg from S. Typhimurium and *S.* Enteritidis in samples from all subjects at each time point will be analysed. Test samples, in triplicate, will be analysed at three dilutions (1:100, 1:4000, 1:160,000), and colour change compared with a standard curve made with calibrated human serum pool included on each assay plate. This pooled serum pool will be collected from high-titre adult donors in Malawi, identified from the STRATAA study (Meiring 2019) validated as the serum standard at GVGH and used across the four sub-Saharan African sites. The anti-O-Ag IgG concentration to S. Typhimurium and S. Enteriditis, in the serum standard was determined at GVGH. Anti-OAg responses will be expressed in ELISA units (EU)/ml. Test sample ELISA units are interpolated from the standard curve. Well-characterized standard OAg from *S.* Typhimurium and *S.* Enteritidis will be used as plate coating antigen. Positive and negative controls are created from the serum standard at set concentrations of 1.41 and 0.69 EU/ml respectively. Standard curves for each plate were assessed by a number of parameters including a minimum r-squared value, minimum (0.5) and maximum (2) optical density readings at 1 EU/ml, and maximum 20% error from expected concentration values from back-calculations (Aruta *et al.*, 2023). BioTek™ 50TS Microplate Washer and BioTek™ 800TS Absorbance Reader will be used for the ELISAs.

***Analysis of clinical sera by SBA***

Serum Bactericidal Activity will be measured using a high-throughput fluorescent assay, which detects bacterial ATP as a readout of viability, developed by members of the VacciNTS consortium ^19^. The assay will be conducted in the presence of full complement repletion with 50% baby rabbit complement. Results will be available on the same day, without the need for overnight incubation or colony counting. The samples from all subjects will be analysed, in triplicate, by SBA assay using *S.* Typhimurium and *S.* Enteritidis wild type strains. Well-characterized cell banks of the test strains will be routinely used for the SBA. The serum standard will be tested on each plate; IC50 of the standard should be in the target range of 3x10 ^3^ and 3x10 ^4^. Validation checks for plates to pass quality control are outlined in [ Aruta *et al*., 2022]. BioTek™ Synergy™ HTX Multi-Mode Microplate Reader will be used for luminescence readings.

All serological laboratory assays (ELISA and SBA) will be standardised throughout the consortium, and quality assurance between sites will be conducted, with the aim of becoming the international standard assays for use in future iNTS clinical trials, thus ensuring highly comparable dataset for modelling approaches across the consortium. Standardized sera has been validated between Malawi and GVGH to ensure reproducibility of both assays. Malawi is the African reference site for the assay. A detailed description of this SBA method has recently been published by Aruta *et al.,* 2022. All sample analysis will be done in Malawi for all sites, including assay transfer of the ELISA and L-SBA assays. The Ghana site will also transfer the assays to their site and will be cross-validated in Malawi.

***Processing and analysis of stool samples***

Briefly, stool samples will be collected in the field in a 30ml universal container in the field and then transported to the laboratory in Blantyre in a cold box at 4–8°C. Subsequently the sample was enriched in selenite broth and incubated overnight at 37°C before aliquoting and freezing at -70°C. Bacterial DNA extraction on the stored selenite samples will be done in batches within 14 days of collection. To avoid any age-related bias, batched analysis will be performed in the order in which the samples were collected. DNA will be extracted from pure cultures using the Omega BioTek Mag-Bind Universal Pathogen kit on the Thermo KingFisher Flex, and then sequenced using Illumina sequencing according to the manufacturer’s instructions.

The remaining selenite was cultured on XLD and incubated at 37oc overnight. Pure colonies with black centres were isolated on XLD and re-cultured on MacConkey (MAC) and Nutrient agar (NA). Oxidase test and API 20E were performed on the pure colonies from NA to identify Salmonella. Salmonella Anti-sera tests were conducted following a positive API20E identification to distinguish between O4 and O9 serovars. Pure salmonella colonies from NA were stored in a commercially purchased microbank vial. A sub-set of the batched stool samples will be tested for myeloperoxidase (MPO) using MPO stool ELISA kits (Immunodiagnostik AG, Germany, Catalogue K6630) as described in the manufacturer’s kit instructions. Stool microscopy will be carried out for the identification of parasites, ova and cysts on the day of sample collection using the Kato-Katz technique.

***Pan-Salmonella PCR***

The ttr primers and probe for the monoplex qPCR were designed and validated by the Federal Institute for Risk Assessment, Berlin, using the *S*. enterica Typhimurium ttr locus DNA sequence (GenBank accession no. AF282268) and methods from Chirambo *et al.* (2020) ^15^. For the qPCR, 200 μl of the top layer from a frozen overnight Selenite F broth stool culture was mixed with 500 μl of PBS.

An optimized PCR protocol from Chirambo *et al.* (2020) ^15^ was used. The 20 μl RT-PCR master mix included 12.5 μl Platinum® Quantitative PCR Super Mix-UDG, 0.10 μl each of forward and reverse primers, 0.10 μl probe (all at 200 nM), 0.05 μl ROX dye (50 nM), and 7.15 μl nuclease-free water. This was added to 96-well plates. Test DNA, positive control DNA (D23580), and negative controls (technical extraction and UV-treated water) were added in triplicates. The qPCR run for 40 cycles on an Applied Biosystems® 7500 Real-Time PCR System, with cycling conditions of 95°C for 1 minute (initial denaturation), 95°C for 15 seconds, 60°C for 30 seconds (annealing/extension), and a final extension at 12°C. The threshold was set in the lag phase. The positive control material is DNA extracted from Salmonella enterica subsp. enterica serovar Typhimurium str. D23580. The negative control material is Nuclease Free water. An assay was deemed valid if the positive controls tested positive and both the technical extraction and assay negative controls tested negative. Test sample cycle threshold (Ct) values were assessed after baseline subtraction, with Ct values of 35 or lower considered positive.

***Salmonella sequencing***

Broth will also be plated onto agar to isolate Salmonella species and determine the range of NTS species causing eNTS episodes. Salmonella positive cultures will undergo Illumina sequencing at the Malawi-Liverpool Wellcome Research Program laboratories to provide phylogenetic analysis to understand NTS molecular epidemiology, which is important for NTS vaccine development and informing other interventions.

***Malaria microscopy and PCR***

All children will have malaria peripheral blood smears (PBS) for microscopy as well as dried blood spot (DBS) and a subset for malaria PCR testing. These will be dried in the field and transported in slide boxes at room temperature to the laboratory in Blantyre, where the films will be read by two independent, blinded trained laboratory technicians, on the day of sample collection.

### Blood cultures

Blood cultures were collected at QECH under standard protocol as described by ( Wilson *et al.*, 2020)

### Statistics and analysis

***Description of statistical methods***

Data analysis will be carried out using the R environment for statistical computation ^20^. We will use changes in SBA along with OAg IgG as the primary measures of immunity in a modelling framework we have already developed, that combines censored regression with linear or cubic splines and allows principled change point analysis ^21, 22^ to identify the age of antibody and SBA nadir for each serovar. We will use this model to describe the impact of age, malaria exposure (MRDT), malnutrition, anaemia and eNTS exposure on SBA and OAg IgG. The models we develop will account for correlations between the paired samples using generalised estimating equations. Regression coefficients and confidence intervals will be calculated for each of the risk factors; malaria RDT positivity, MUAC, haemoglobin, and adjusted for season, poverty and sanitation. Percentage change in IgG and SBA between paired samples will be calculated, plotted against age and compared by season, using generalised linear models. Number of seroconversions by age will be calculated, with the seroconversion defined by fold-increases in IgG or SBA. The sero-incidence rates will be compared across risk factor groups and by season. Finally, additional contrasting datasets (cross-sectional sample sets from vacciNTS sites in Burkina Faso, Ghana and Kenya) will allow us to compare immunological markers in different settings.

As an exploratory analysis, mechanistic mathematical models will be developed to identify a population correlate of protection using acquisition of IgG and SBA by age with rates of invasive NTS disease by age. Additional exploratory analyses will include impact of RTS,S malaria vaccination on the development of immune response to NTS, graphical models (Bayesian networks, structural equation models or Gaussian copula graphical models), to interrogate the complex relationships between the immunological and environmental variables; and geospatial analyses of the GPS-mapped subject and household data.

### Ethical and regulatory considerations

***Approvals***

The protocol, informed consent form, participant information sheet and any proposed advertising material will be submitted to the sponsor (University of Liverpool) and the University of Malawi College of Medicine Research Ethics Committee (COMREC), and University of Liverpool (UoL) Ethical Review Board for written approval.

○ COMREC reference approval number P.07/20/3092
○ University of Liverpool Sponsorship reference number: UoL001562
○ University of Liverpool ethics reference number: 7923

The Investigator will submit and, where necessary, obtain approval from the above parties for all substantial amendments to the original approved documents.

Each site (Malawi, Kenya, Burkina Faso and Ghana) will obtain their own local ethics approval.

### Sponsor

The University of Liverpool will act as Sponsor for this study. It is recognised that as an employee of the University the Chief Investigator has been delegated specific duties, as detailed in the Sponsorship Approval letter.

***Insurance***

The University of Liverpool will be the Sponsor of the study and has specialist indemnity and insurance cover with Marsh UK Ltd which would operate in the event of any participant

## Data Availability

No data are associated with this article. Open Science Framework: SAiNTS protocol documents, laboratory and clinical SOPs, data management plan and analysis code https://doi.org/10.17605/OSF.IO/FUE3T ^6^ Analysis code available from: https://github.com/helda42/SAINTS_protocol_sample_size Archived analysis code at time of publication: https://doi.org/10.5281/zenodo.7251872 ^18^ License: MIT
