# Supplementary material for: Understanding the epidemiology of iNTS disease in Africa in preparation for future iNTS- vaccine studies in endemic countries: Seroepidemiology in Africa of iNTS (SAiNTS) Study Protocol: Malawi site [Version 9.0]
Source: Wellcome Open Res. 2024 Aug 21;8:27. Originally published 2023 Jan 18. [Version 2] doi: 10.12688/wellcomeopenres.18054.2 (PMC11344207; doi:10.12688/wellcomeopenres.18054.2)
Supplement: Peer review history

# Reviewer report: Reviewer response for version 2

**DOI:** 10.21956/wellcomeopenres.23635.r94409

**Published:** 2024-09-16

**Authors:** Aaron P White

**Affiliations:** 1 Vaccine and Infectious Disease Organization (VIDO), University of Saskatchewan, Saskatoon, Canada

**Figure 3 **- I don't understand why some boxes are greyed out. Perhaps this could be explained in the Figure legend.

**Table 3** - GANTT chart is empty - is that on purpose or a mistake? NO other questions or proposed changes.

Is the study design appropriate for the research question?

Yes

Is the rationale for, and objectives of, the study clearly described?

Yes

Are sufficient details of the methods provided to allow replication by others?

Partly

Are the datasets clearly presented in a useable and accessible format?

Yes

Reviewer Expertise:

Salmonella genetics, transmission, vaccine development - Bacterial genomics

I confirm that I have read this submission and believe that I have an appropriate level of expertise to confirm that it is of an acceptable scientific standard.

# Reviewer report: Reviewer response for version 2

**DOI:** 10.21956/wellcomeopenres.23635.r94408

**Published:** 2024-08-23

**Authors:** Emma Slack

**Affiliations:** 1 Institute of Microbiology, Department of Biology, ETH Zurich Foundation, Zürich, Zurich, Switzerland; 2 Sir William Dunn School of Pathology, University of Oxford (Ringgold ID: 6396), Oxford, England, UK

The information on serology analysis is now clear.

Is the study design appropriate for the research question?

Yes

Is the rationale for, and objectives of, the study clearly described?

Yes

Are sufficient details of the methods provided to allow replication by others?

Partly

Are the datasets clearly presented in a useable and accessible format?

Not applicable

Reviewer Expertise:

Salmonella O-antigen biology, glycoconjugate vaccine development, non-Typhoidal Salmonellosis

I confirm that I have read this submission and believe that I have an appropriate level of expertise to confirm that it is of an acceptable scientific standard.

# Reviewer report: Reviewer response for version 1

**DOI:** 10.21956/wellcomeopenres.20021.r63052

**Published:** 2024-08-30

**Authors:** Virginie Stévenin

**Affiliations:** 1 Department of Cell and Chemical Biology, Oncode Institute, Leiden University Medical Center (LUMC), Leiden, The Netherlands

**Summary:**

 The authors provide a detailed study protocol aiming at investigating the acquisition of immunity to NTS and understanding the epidemiology of enteric NTS infection in children 0-5 years of age. In particular, the interactions between risk factors (poverty, sanitation, climatic conditions, immune status of malaria, malnutrition, and anemia), NTS infection, and the production of antibody and bactericidal activity will be assessed.

 For this, 2500 children 0-5 years of age will be recruited for data and sample collection via two-stage visits and a subset of children will have extra follow-up visits.

**Comments:**

 This study protocol is detailed and well-written. The background is properly introduced. The broad, specific, and exploratory objectives are clearly presented.

**Request:**

 I would like the authors to include information or comment about the following point:

 The authors explained in "The acquisition of natural systemic immunity" paragraph that the nadir of functional humoral immunity occurs after the waning of maternal antibodies.

 As breastfeeding and transfer of maternal antibodies possibly protect the children and modify antibody and SBA titers, how is this taken into account in the protocol design? Is information regarding the duration and time since breastmilk sewerage collected?

**Minor edits:**

- In the methods, timing in months is provided in both digits and letters in the same sentences. Please homogenize.
- Salmonella should always be written with a capital S.
- Could the authors precise what they mean by "deemed clinically unstable"?
- In the "Change in SBA due to eNTS" section, the loss of follow-up is estimated at 20%, but it is previously written "statistical power calculations have assumed a further 10% drop out for the second visit". Does this refer to dropouts at different stages?
- I agree with the Reviewer Report 21 February 2023 that the sample size should be consistent throughout the text (2000 or 2500?).
- Has the unpublished work from T. Nyirenda mentioned in the text been published since the last version of the protocol? If yes, please update the text with the appropriate reference.

Is the study design appropriate for the research question?

Yes

Is the rationale for, and objectives of, the study clearly described?

Yes

Are sufficient details of the methods provided to allow replication by others?

Yes

Are the datasets clearly presented in a useable and accessible format?

Yes

Reviewer Expertise:

Salmonella infection, host-pathogen interaction

I confirm that I have read this submission and believe that I have an appropriate level of expertise to confirm that it is of an acceptable scientific standard.

# Reviewer report: Reviewer response for version 1

**DOI:** 10.21956/wellcomeopenres.20021.r61031

**Published:** 2024-03-27

**Authors:** Aaron P White

**Affiliations:** 1 Vaccine and Infectious Disease Organization (VIDO), University of Saskatchewan, Saskatoon, Canada

The authors present an extensive plan for determining robust antibody correlates of protection for invasive NTS disease. This is still a huge burden in SSA and more information is needed to inform public health measures for control of these infections. There are many factors to consider and the authors have done a good job to try to account for the main risk factors / influences as it relates to iNTS infections in children aged 0-5 years.  

 The inclusion / exclusion criteria are clearly laid out as well as the different factors that the authors will be considering – malaria, malaria vaccine exposure, Salmonella culture and/or PCR positivity, malaria-related anemia, malnutrition. The authors have also done a good job with their power calculations for each factor – taking into account potential drop-out rates from one sampling to another, and then relating this to meaningful differences that can be potentially detected between groups. The authors have clearly thought out the existing problems surrounding iNTS infections and general health, which is based in clinical experience. I am hopeful that many important trends and correlations can be determined from this 3 year study.

 Regarding methodology, the authors did a good job explaining how the sampling would be performed. However, the description of the antibody (O-antigen specific) tests and serum bactericidal activity assays were extremely brief. In my opinion, more information should be provided for each. For example, although the protocols are described in referenced papers, the procedures are always tweaked to work with different sample types and it would benefit to provide more detail. i.e., positive controls should be defined, the parameters of measurement, expected range, instruments used, etc.  For Salmonella culturing, what media will be used and how will the stool samples be treated?, for Salmonella PCR, I realize it is a validated test but what are the parameters of measurement and what is a ‘positive’ result being compared to? How will whole genome sequencing of cultured Salmonella strains be performed? I don’t think the assembly details are important, but what sequencing platform(s) will be used and where will the experiments be performed? The authors may feel like some of these details are trivial or not important, but their inclusion can help others when they are planning other surveillance studies around the world.  

 The section “Sample size simulations for serosurvey” I didn’t understand well and needs more clarity: 1000 single samples (200 in each of 5 age bands) – I took this to mean age 1, age 2, age 3, age 4 and age 5 – is this correct? It should be clarified what the authors mean by “age band”

 Also, how does this minimum sample size for each site correspond to the 2000 stool samples and 4000 paired blood samples mentioned in the “Prospective community cohort” section?

 In Figure 3, one of the factors included is “Rainfall and Temperature”. I wondered if the authors could expand on this a little bit – how are these factors thought to contribute to iNTS infection rates? Is this related to increased mobilization of potential environmental reservoirs of Salmonella? Or are there changes in human behaviour that would bring more exposure?

 A few typos were observed:

  

1. First paragraph of introduction – the authors introduce the case fatality rate (CFR) but a few sentences later CRF is referenced – which one is it?
2. Figure 1 – Phase 1 clinical “STUFY” is in box #2
3. Figure 2B – what does the lower case “a” refer to above the SBA graph?
4. The Figure legend to 2B didn’t make sense – “Age distribution of immunity to anti S. typhimurium LPS antibodies” –the word immunity doesn’t seem to fit here
5. EpiMal is referred to on Page 5, but later is written as EPIMAL – the authors should be consistent throughout the manuscript when referring to this group

Is the study design appropriate for the research question?

Yes

Is the rationale for, and objectives of, the study clearly described?

Yes

Are sufficient details of the methods provided to allow replication by others?

Partly

Are the datasets clearly presented in a useable and accessible format?

Yes

Reviewer Expertise:

Salmonella genetics, transmission, vaccine development - Bacterial genomics

I confirm that I have read this submission and believe that I have an appropriate level of expertise to confirm that it is of an acceptable scientific standard, however I have significant reservations, as outlined above.

# Reviewer report: Reviewer response for version 1

**DOI:** 10.21956/wellcomeopenres.20021.r66911

**Published:** 2024-03-27

**Authors:** Emma Slack

**Affiliations:** 1 Institute of Microbiology, Department of Biology, ETH Zurich Foundation, Zürich, Zurich, Switzerland; 2 Sir William Dunn School of Pathology, University of Oxford (Ringgold ID: 6396), Oxford, England, UK

This protocol details a major prospective study to be carried out across multiple countries assessing serology and culture-based observations of iNTS disease. The scope of the clinical cohort, particularly as this focuses on children under the age of 5, and the proposed sampling schemes are well thought-out and justified. The background and aims are clear and the presentation of power calculations and data simulations shows excellent attention to detail on the clinical aspects of the project.

 To improve the chances to achieve the desired scientific outcome, I would have some very detailed comments on the methods being used to study iNTS serology:

 1) The authors propose to focus on the Salmonella O-antigen-specific IgG response. The rationale for this is good, as there is some correspondence to protection. However, it should be noted that the group A, B, D and E Salmonella O-antigens (i.e. most of the strains commonly infecting humans) are rather closely related structures, sharing a mannose-rhamnose-galactose backbone with the only difference being the identity of the repeat unit di-deoxy-hexose side-branch. Correspondingly there is quite some cross-reactivity between IgG responses raised against the O antigens of serovars Enteritidis, Paratyphi, Typhi and Typhimurium.

 For Example: This is generally not sufficient to generate cross-protection but could be confounding when using serology to predict epidemiology. Have the authors considered including ELISAs specific for proteinaceous virulence factors that would distinguish iNTS from Typhimurium eNTS? I am not certain whether this is possible, but at least the confounder of variable cross-reactivity needs to be carefully considered in interpretation of the data.

 2.) It is also clear whether the ELISA systems proposed are home-made or commercial, and if commerical who the supplier is. As there are differences between kits in terms of sensitivity/specificity, this is important information for anyone trying to replicate the study.

 3.) Note that the complete names of the bacteria you are tracking are *Salmonella enterica subspecies enterica *serovar Typhimurium/Typhi/Enteritidis. This is a common confusion in the field related to common usage of the contracted forms, but the designations Typhimurium/Typhi/Enteritidis are not part of the species names and are generally written non-italicized, with a capitalized first letter to indicate this.

Is the study design appropriate for the research question?

Yes

Is the rationale for, and objectives of, the study clearly described?

Yes

Are sufficient details of the methods provided to allow replication by others?

Partly

Are the datasets clearly presented in a useable and accessible format?

Not applicable

Reviewer Expertise:

Salmonella O-antigen biology, glycoconjugate vaccine development, non-Typhoidal Salmonellosis

I confirm that I have read this submission and believe that I have an appropriate level of expertise to confirm that it is of an acceptable scientific standard, however I have significant reservations, as outlined above.

## References

[1] : Structural diversity in Salmonella O antigens and its genetic basis. *FEMS Microbiol Rev* .2014;38(1) : 10.1111/1574-6976.12034 56-89 10.1111/1574-6976.12034 23848592

# Reviewer report: Reviewer response for version 1

**DOI:** 10.21956/wellcomeopenres.20021.r66915

**Published:** 2023-10-13

**Authors:** Shifeng Wang

**Affiliations:** 1 Department of Infectious Diseases and Immunology, College of Veterinary Medicine, University of Florida, Gainesville, USA

This protocol aims to investigate the multifaceted impact of various risk factors on invasive Non-Typhoidal *Salmonella* (iNTS) infection and acquired immunity, specifically within the context of a phase 4 malaria vaccine cluster trial in Chikwawa, Malawi. The study will involve collecting paired samples from 2500 children aged 0-5 years for 2 visits over a 3-month interval. These samples will be meticulously tested for *Salmonella* through stool culture, serum O-antigen specific antibodies against *S. *Typhimurium and *S.* Enteritidis, serum bactericidal antibody (SBA) function, and signs of intestinal inflammation. Concurrently, data on associated risk factors such as malaria, anaemia, malnutrition, socioeconomic status, environmental factors (temperature, rainfall), and water and sanitation conditions will be gathered. In addition, a subset of individuals with invasive NTS disease will be closely monitored for up to 12 months to gain comprehensive insights into disease progression and outcomes.

 The title is about the seroepidemiology in Africa of iNTS. The protocol primarily discusses the specifics of one of 4 African sites, the Malawi site within the context of a malaria vaccine trial.  A consideration is whether the other sites will share a similar vaccine setting. This necessitates a brief introduction to each selected country, highlighting their differences and similarities with the Malawi site.

 There is discrepancy about the sample sizes in abstract, study design and sample size. Please clarify the sample size is 2000 or 2500. 

 In the section of  Case-control study, there is a lack of sample size information.

 In the Prospective Community Cohort section, will the treatment be recorded and analyzed? Will this tracking continue for 12 months? Considering that some treatments may involve antibiotics, information about antibiotic usage should be included during the visits.

 The protocol lacks a description of the process for sample processing, such as method of isolating *Salmonella *from stool samples, conducting blood cultures, and utilizing DNA sequencing services. Is there a specific protocol detailing how the samples will be processed – whether immediately upon collection or frozen for batch processing, especially concerning stool and serum samples? Additionally, clarity is needed on the processing sites; will the processing take place in Malawi or will samples need to be shipped to England? Moreover, are other sites expected to process samples on-site, or will they also need to ship them to either Malawi or England?

 In the section of Data Availability, the authors have uploaded many documents to OSF. It should be stated here. Although no data is generated in this protocol, a data management plan for data generation, storage, and sharing should be established.

 Minor

 In the section of Introduction, Line 10, CRF seems should be CFR.

 In the whole protocol, the authors should ensure that the term " *Salmonella*" is correctly formatted. ' *Salmonella*' should be italicized, while 'Typhi,' 'Enteritidis,' and 'Typhimurium' should not be italicized as they represent specific serovars. *P. falciparum* should be italicized in Table 2.  

 In the section of Enteric exposure to iNTS, a reference for the statement 'Quantitative PCR using a pan-Salmonella marker (TTR, tetrathionate reductase gene) was more sensitive than culture for detection' should be included.

 Figure 2. The reference for the data should be included.

 TABLE 1, ROW 2 should include *S.* Enteritidis LPS

 Page 8. Line 5, children should be deleted.

Is the study design appropriate for the research question?

Yes

Is the rationale for, and objectives of, the study clearly described?

Yes

Are sufficient details of the methods provided to allow replication by others?

Partly

Are the datasets clearly presented in a useable and accessible format?

Yes

Reviewer Expertise:

Vaccinology, Salmonella

I confirm that I have read this submission and believe that I have an appropriate level of expertise to confirm that it is of an acceptable scientific standard, however I have significant reservations, as outlined above.

# Reviewer report: Reviewer response for version 1

**DOI:** 10.21956/wellcomeopenres.20021.r54266

**Published:** 2023-02-21

**Authors:** Clare L. Cutland

**Affiliations:** 1 African Leadership in Vaccinology Expertise, Faculty of Health Sciences, University of the Witwatersrand, Johannesburg, South Africa

This protocol is for a prospective study, to be conducted in an EPIMAL (RTS,S/ AS01 study) phase 4 trial population in Chikwawa, Malawi (and Kenya, Ghans & Burkina Faso?). Two visits, 3-months apart will be conducted on 2500 children aged 0-5 years of age to collect blood to assess age-stratified acquisition of lipopolysaccharide O-antigen (IgG) and serum bactericidal activity to main serovars causing invasive non-typhoidal salmonella. Additionally, stool samples will be collected at one timepoint (enrolment) to culture for NTS and pan-Salmonella PCR (exposure).

 iNTS has bimodal age distribution, affecting mainly people living with HIV and young children. Children with malaria, anaemia & malnutrition are highly susceptible to iNTS.

 There is currently no licensed vaccine, but 3 vaccines are in clinical trials.

 Methods: 2500 children will be recruited with parental consent by field teams visiting EPIMAL clusters. Data and sample collection and processing will be standardized and sample processing will be supported (lab training and study support) by GVGH.

**General comment**: well-written, detailed protocol, but needing a few amendments to clarify the following:

- Is same protocol being used for Malawi, Kenya, Ghana & Burkina Faso? Background & methods sections focus on Malawi, but objectives include all 4 countries. Please include all 4 countries in background and methods.
- Please clarify sample size- 2000 or 2500? Also, is this sample size for Malawi only, or across all 4 country sites?
- Brief schedule of events table will be a welcome addition.

**Detailed comments:**

- Introduction, row 9- used CRF instead of CFR- please correct.
- Methods section mentions 2000 children, but abstract and sample size calculations sections refer to 2500 children. Please correct.
- Standardise Epimal/ EPIMAL throughout document.
- In ‘Longitudinal cohort and weekly stool sampling’ section, top of pg 8: please revise this sentence starting ‘We will carry out weekly…’- looks like ‘children’ repeated.
- ‘ *Salmonella typhi’* is scientific name. Please capitalize ‘S’ and italicize. (Case control study section- please check whole protocol).
- Please clarify: for Salmonella sequencing, are you referring to iNTS cases only, or all iNTS, plus stool sample isolates too?
- A ‘schedule of events’ table would be useful.
- Please include details of data collection platform to be used- paper or electronic? If electronic, which platform?
- Use of OSF as a document storage portal is good, and easily accessible. Suggest organize OSF project into folders for ‘protocol’, ‘ICFs’, ‘Ethics’, ‘CRF’ etc.

Is the study design appropriate for the research question?

Yes

Is the rationale for, and objectives of, the study clearly described?

Yes

Are sufficient details of the methods provided to allow replication by others?

Partly

Are the datasets clearly presented in a useable and accessible format?

Not applicable

Reviewer Expertise:

Clinical vaccine trialist, vaccinology

I confirm that I have read this submission and believe that I have an appropriate level of expertise to confirm that it is of an acceptable scientific standard, however I have significant reservations, as outlined above.
